# Cut! that’s a wrap: regulating negative emotion by ending emotion-eliciting situations

**DOI:** 10.3389/fpsyg.2014.00165

**Published:** 2014-02-28

**Authors:** Lara Vujovic, Philipp C. Opitz, Jeffrey L. Birk, Heather L. Urry

**Affiliations:** Department of Psychology, Tufts University, MedfordMA, USA

**Keywords:** situation selection, situation modification, attentional deployment, process model, emotion regulation, SOC-ERpt

## Abstract

Little is known about the potentially powerful set of emotion regulation (ER) processes that target emotion-eliciting situations. We thus studied the decision to end emotion-eliciting situations in the laboratory. We hypothesized that people would try to end negative situations more frequently than neutral situations to regulate distress. In addition, motivated by the selection, optimization, and compensation with ER framework, we hypothesized that failed attempts to end the situation would prompt either (a) greater negative emotion or (b) compensatory use of a different ER process, attentional deployment (AD). Fifty-eight participants (18–26 years old, 67% women) viewed negative and neutral pictures and pressed a key whenever they wished to stop viewing them. After key press, the picture disappeared (“success”) or stayed (“failure”) on screen. To index emotion, we measured corrugator and electrodermal activity, heart rate, and self-reported arousal. To index overt AD, we measured eye gaze. As their reason for ending the situation, participants more frequently reported being upset by high- than low-arousal negative pictures; they more frequently reported being bored by low- than high-arousal neutral pictures. Nevertheless, participants’ negative emotional responding did not increase in the context of ER failure nor did they use overt AD as a compensatory ER strategy. We conclude that situation-targeted ER processes are used to regulate emotional responses to high-arousal negative and low-arousal neutral situations; ER processes other than overt AD may be used to compensate for ER failure in this context.

## INTRODUCTION

Emotional responses are often useful in our everyday life, but can, depending on the context, be inappropriate. Imagine being sick in a hospital, undergoing an unpleasant medical procedure that is your only option to get healthy again. As a response to this situation, you might feel afraid, your heart might start racing, and you might have the urge to leave the room. However, in order to meet your goal for this situation (getting healthy), you reduce your distress during the procedure by staying in the room, taking a couple of deep breaths, distracting yourself by counting floor tiles, reminding yourself that the outcome is worth it, holding the hand of your significant other, or all of the above. This is just an illustration of when and how we routinely regulate our emotions based on the context we are in (Gross et al., 2006).

As the example above may suggest, there are many ways in which we can regulate our emotions. One particularly useful and influential framework for organizing these many ways of regulating our emotions is the process model of emotion regulation (ER; Gross, 1998). According to this model, five families of ER processes each target one of four components of the emotion generative cycle (Gross and Thompson, 2007), the situation, attention to the situation, appraisals of the situation, or the multisystem response. That is to say, we can select the situations we put ourselves in based on the emotions we anticipate experiencing (“situation selection”). Once in a situation, we can modify that situation to alter our emotional experience (“situation modification”). These two ER families target the situation component of the emotion generative cycle. We can attend to different aspects of the situation (“attentional deployment”), reappraise it (i.e., generate a new meaning; “cognitive change”), and/or finally, directly alter our experiential, expressive, or bodily response to the situation (“response modulation”). The latter three ER families target the attention, appraisal, and multisystem response components of the emotion generative cycle, respectively.

Much has been learned about the frequency and success with which attentional deployment, cognitive change (particularly cognitive reappraisal, CR), and response modulation (particularly expressive suppression) are used. On the frequency side, when asked to describe episodes of ER usage in the preceding 2 weeks, participants frequently report using attentional deployment (in 39% of the episodes), cognitive change (in 33% of the episodes, most of which were instances of CR), and response modulation (in 53% of the episodes, most of which were instances of expressive suppression; Gross et al., 2006). In addition, in a performance-based laboratory task designed by Jackson et al. (2000), 56% of participants reported using cognitive ER strategies and 40% reported using attentional deployment when asked to decrease their emotional responses to negative pictures. On the success side, attentional deployment, CR, and expressive suppression have all been shown to be effective in altering emotion in accordance with the regulatory goal (e.g., Gross and Levenson, 1993; Urry, 2010; see Webb et al., 2012, for a recent meta-analysis). Moreover, as reviewed by Gross et al. (2006), CR is effectively used to decrease unpleasant emotional experiences without significant interpersonal, cognitive, and physiological costs. Expressive suppression, by contrast, is effective in down-regulating expressive behavior, but has little effect on experience of unpleasant emotions. In fact, more frequent usage of expressive suppression has been linked to decreased well-being, emotional and social functioning (Badcock et al., 2011; Gross et al., 2006), at least in Western cultures.

In contrast to the families of ER described above, to our knowledge less is currently known about the frequency and success of situation selection and situation modification, at least with respect to their coverage in the ER literature. In fact, there are so few studies available that these two families of ER were not included in Webb et al. (2012) meta-analysis. That being said, substantial evidence about goal-oriented approach-avoidance behavior comes from the literature on coping. In this literature, approach strategies include actively thinking about the situation in different ways while avoidant strategies include denial of threat and getting away from the unpleasant situation (Ebata and Moos, 1991).

In their review, Mullen and Suls (1982) found that avoidant strategies are generally effective short-term solutions, while approach strategies are more beneficial for long-term goals. For instance, one can easily appreciate that avoiding certain situations, such as going to a party or giving a speech in public, will be useful in the short-term if it prevents acute anxiety. However, this sort of avoidance may be detrimental in the long-term if it prevents one from habituating to the situation and learning that the worst-case scenario usually does not happen (e.g., you will not be ostracized at the party or booed off stage during the speech; Campbell-Sills and Barlow, 2007). Subsequently, avoidant strategies have been shown to be useful for reducing stress and preventing overwhelming anxiety, while approach strategies have been shown to facilitate taking appropriate action and assuming more control over the situation (Roth and Cohen, 1986).

While approach and avoidance strategies have been shown to be beneficial, research suggests that there can be costs associated with using both. For example, approaching the situation in which there is little room for change can lead to increased distress and worry (Roth and Cohen, 1986). Moreover, clinical evidence suggests that using avoidance strategies chronically or inflexibly can maintain psychopathology, as is the case in avoidant personality disorder and social anxiety disorders (Campbell-Sills and Barlow, 2007). Additionally, usage of avoidant strategies has been linked to depression (Herman-Stabl et al., 1995). Overall, it seems that benefits and costs linked with avoidance and approach are dependent on the context in which they are used.

It is important to note that in the coping literature, approach and avoidance strategies are defined in ways that conflate the ER processes involved. For example, viewed from within the process model of ER, avoidance coping involves both situation selection (getting away from the unpleasant situation) and cognitive change (denial of threat; Ebata and Moos, 1991). And yet, these families of ER are rather different. Situation selection, according to the process model of ER, refers to behavioral strategies people can use to put themselves into (or take themselves out of) particular situations. This is distinct from cognitive change, which refers only to as the cognitive act of appraising situations differently. While it makes good sense to consider these strategies, which may regularly go hand in hand in daily life, together, it may also be scientifically useful to isolate strategies that target the situation component of the emotion generative cycle (situation selection, situation modification) from strategies that target the appraisal component (cognitive change).

Studies reviewed above seem to indicate that situation-targeted ER strategies have potentially powerful effects, yet they have not been extensively studied within the context of the process model of ER. There have been some efforts in this direction, of course. For example, Gross et al. (2006) conducted semi-structured interviews about daily experiences of ER. These interviews revealed that people do not report using situation selection/modification often. However, while this study represented an important step and interview data are very informative, they are limited to telling us about the strategies people are aware of and/or willing to report using. Considering the limitations of retrospective self-report, it is possible that the actual frequency with which people use certain ER strategies could be different. For example, people may be unaware that they chose to enter certain situations because of how those situations were expected to make them feel or simply not remember the situations they chose *not* to enter. Because of this, it is important to examine situation-targeted ER strategies using other methods as well.

One example of an objective method of examining these strategies is reflected in the pioneering effort of Rovenpor et al. (2013). These authors developed the Affective Environment task to measure situation selection. In this task, participants spent several minutes in a room in which there were opportunities to engage with valenced stimuli, consisting of websites, television news stories, and hard-copy articles. Using this task, Rovenpor et al. (2013) found that older adults with ER self-efficacy and general control beliefs chose to engage with fewer negative stimuli, while their younger adult counterparts chose to engage with more negative stimuli. These results suggest that people select situations as a function of the emotional potential of those situations and this choice is moderated by individual differences (e.g., age, self-efficacy, control beliefs).

The reasonable assumption of the above findings is that people approach or avoid negative situations in part because of how unpleasant those situations are likely to be. Of course, feeling negative is not the only feeling that is likely to motivate situation selection. A positive feeling such as interest can also motivate situation-targeted strategies. Lang et al. (1993) designed a task in which participants viewed and rated negative, neutral, and positive pictures. After completing questionnaires, participants viewed these pictures again along with a set of new pictures to facilitate recognition memory testing. Importantly, after making their recognition decision, participants had the option to press a key to make the pictures disappear from the screen. The authors showed that longer picture viewing time was positively correlated with greater ratings of interest, meaning that people tended to look longer at interesting stimuli. Thinking of viewing time as an indicator of situation selection (i.e., participants remained in interesting situations longer), these results suggest that interest (or the lack thereof, also known as boredom) may also motivate the decision to use situation-targeted ER strategies.

The selection, optimization, and compensation with emotion regulation (SOC-ER) framework suggests that we select and optimize ER strategies, and if they are not successful, we might compensate by employing other ER strategies (Urry and Gross, 2010; Opitz et al., 2012). Imagine again that you’re in the hospital, about to undergo that unpleasant medical procedure, but before you do, the nurse has to draw your blood. You were not aware of this, and you start getting nervous. You try to end this situation (and thus your distress) by asking the nurse to draw the blood later (situation selection/modification). The nurse responds that she needs to do it right now, per doctor’s orders. So, while she draws your blood, you look away from the needle to the flowers in your hospital room (attentional deployment) in order to reduce your distress. As illustrated in this example, when your attempt to end the situation (attempting to postpone the blood draw) failed to regulate your negative emotions, you tried to compensate for this failure using attentional deployment instead (looking at the flowers).

Consistent with the idea of compensation, there is evidence suggesting that people use multiple ER strategies, perhaps to make themselves feel better. For instance, Aldao and Nolen-Hoeksema (2013) found that 65% of participants reported using two or more ER strategies while watching a short disgust-eliciting video clip. In addition, Wolgast et al. (2011) provided evidence suggesting that people who failed to reduce negative emotional experiences using CR turned to avoidance, while people who used acceptance turned to avoidance less. In their experiment examining CR to increase and decrease the negative emotions triggered by pictures, van Reekum et al. (2007) demonstrated via eye tracking that participants deployed their attention toward and away from emotion-triggering information in the pictures and that the extent to which they did so explained significant variance in reappraisal-related neural activation. More recently, Bebko et al. (2011) found that attentional deployment is used in the context of both CR and expressive suppression, further indicating that ER strategies are employed concurrently. Collectively, these examples hint that people use multiple strategies to regulate their emotions and, possibly, that they do so in part to compensate for ER failure. However, this conclusion is speculative. Needed are studies that provide empirical confirmation using methods that directly assess the extent to which people use alternative ER strategies when their original ER strategies fail to have the intended effect.

In sum, there is empirical and clinical evidence suggesting that situation-targeted ER strategies are used in everyday life and that inappropriate usage of these strategies has been linked to behavioral and mental disorders. Furthermore, there is evidence suggesting that people tend to use multiple ER strategies to improve their emotional experience, which might in part reflect the attempt to compensate for unsuccessful ER outcomes. It is still unclear how often people actually use situation-targeted ER strategies, and under which conditions they choose to do so. It is also unclear what happens when situation-targeted ER strategies fail to have the intended effect on our emotions. Answering these questions will lead us to a more complete understanding of the complex mechanisms proposed in the process model and the SOC-ER framework. Furthermore, understanding the contexts in which situation-targeted ER strategies are used and with what degree of success will help inform treatments for the behavioral and mental disorders linked to maladaptive usage of these strategies.

The purpose of the present study was to assess how often people choose to end negative situations if given the opportunity, as well as their stated reasons for doing so. In addition, we sought to determine whether people engage in compensatory ER strategies when attempts to end these situations fail. To address these issues, participants viewed a set of negative and neutral pictures, and were told that they could press a button if they wished to stop looking at the picture. On half of those trials, the button press succeeded; the picture was replaced by a blank screen for the remaining picture presentation time. On the other half of those trials, the button press failed; the picture remained on screen for the remaining picture presentation time. We thus manipulated outcome: whether or not the attempt to end the situation was a success or failure on trials in which participants pressed the button. We collected the percentage of button presses and reaction time (RT) to press the button to index the frequency and rapidity of attempts to end the situation, respectively. We also recorded eye gaze data using eye tracking to index overt attentional deployment, and self-report arousal ratings, corrugator activity, heart rate (HR), and skin conductance to index emotional responding.

We hypothesized that, in order to regulate their emotional response, people would attempt to end the situation (press the button) more often and faster on negative trials than on neutral trials (Hypothesis 1). We further hypothesized that they would report doing so because of distress more so in the negative than in the neutral condition (Hypothesis 2). Pursuant to deciding to end the situation, we also hypothesized that one of two alternatives might be observed. On the one hand, for the negative condition more so than the neutral condition, people may experience more negative emotion in the failure condition relative to the success condition, as reflected in self report and changes in physiology (Hypothesis 3a). On the other hand, they may compensate by engaging in other ER strategies. In this case, negative emotion would be similar in the success and failure conditions, but there would be greater compensatory ER (i.e., looking away from emotional content in the pictures) in the failure condition relative to the success condition (Hypothesis 3b). For this compensatory hypothesis, we focused specifically on attentional deployment as reflected in eye tracking. Attention is a particularly important component of ER processes (Wadlinger and Isaacowitz, 2011). In addition, in this picture-viewing context, looking away from the emotional content of the pictures has been demonstrated to occur in studies examining other forms of ER (van Reekum et al., 2007; Bebko et al., 2011). Attentional deployment would, thus, be one obvious and potentially common alternative ER strategy when one’s attempt to end the situation fails.

## MATERIALS AND METHODS

### PARTICIPANTS

Sixty-two Tufts undergraduates participated in the study. 41 of these participated in exchange for course credit. 21 were recruited though advertisements posted on the internet (http://www.tuftslife.com) and received $10 per hour as compensation. Because we anticipated some loss of data due to equipment problems, artifacts, and extreme or missing values, this sample size was determined with the goal to have approximately 50 useable observations in hypothesis testing. The data for one participant were removed from analyses because the participant misunderstood the task. Another participant was inattentive to the task and talkative and skipped many trials. Eye tracking data were not available for two additional participants due to equipment failure.

Fifty-eight participants were included in our final sample. Of these, 39 participants were female (67%), 34 were White (59%), 10 were Asian (17%), 9 were African American (16%), 1 was Native Hawaiian (2%), and 3 were multiracial (4%), and one person did not wish to disclose this information (2%); 5 of our participants considered themselves to be of Hispanic or Latino origin (8.6%). The age range was from 18 to 26 years (*M* = 19.36 years, *SD* = 1.53 years). All procedures were approved by the Social, Behavioral, and Educational Research Institutional Review Board at Tufts University. Participants provided written informed consent before participating.

### MATERIALS AND PROCEDURES

#### Stimuli

Participants viewed a set of digital color pictures (800 pixels × 600 pixels) selected from the International Affective Picture System (Lang et al., 2008). We selected 30 negative pictures on the basis of normative data across men and women to be highly unpleasant (*M* = 2.69, *SD* = 0.72, on a scale ranging from 1 to 9 where 9 is *completely happy*), and highly arousing (*M* = 5.61, *SD* = 0.96, on a scale ranging from 1 to 9 where 9 = *completely aroused*). In addition, we selected 30 neutral pictures to be neither pleasant nor unpleasant (*M* = 5.23, *SD *= 0.41), and low in arousal (*M* = 3.51, *SD* = 0.54)^1^.

#### Picture task

Participants viewed the above set of 60 pictures, evenly distributed across two blocks. The order in which the 60 pictures were presented was randomized for each participant. Participants were told in advance that they could press the space bar whenever they wished to stop looking at a picture. They were further told that sometimes the picture would go away when they pressed the button (success trials) and sometimes it would remain on screen (failure trials); they were not told that there would be a roughly 50:50 ratio of success to failure trials. The picture task was presented using E-Prime 2.0 (Psychology Software Tools, Pittsburgh, PA, USA).

**FIGURE 1:**
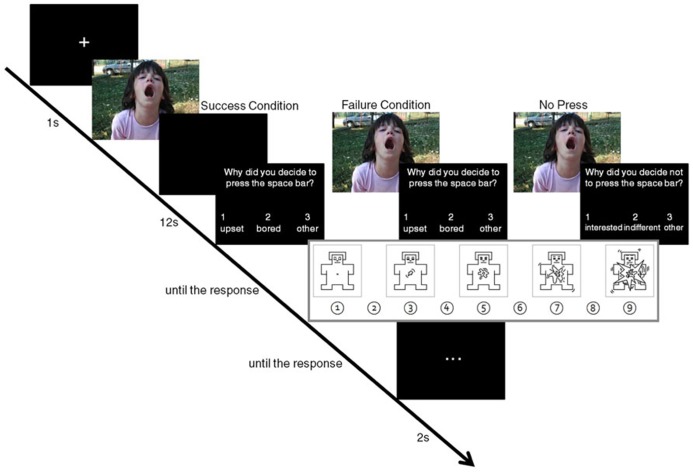
**Trial structure for the picture task.** Note that when they pressed the space bar, participants were offered the following full-sentence reasons: “I was upset by the picture,” “I was bored by the picture” and “Other.” If they did not press the space bar, they were offered the following reasons: “I was interested in the picture,” “I was indifferent to the picture” and “Other.” These full descriptions are not presented in the figure proper due to space constraints; instead we use the terms “upset,” “bored,” “interested,” “indifferent,” and “other.” While we used IAPS pictures for this task, the example picture in this figure is from one of the author’s private collection.

The trial structure for the picture task is presented in **Figure 1**. All trials began with a white fixation cross presented in the center of a black screen for 1 s. This was followed by the presentation of a picture for up to a total of 12 s. If participants chose not to press the space bar, the picture would stay on the screen for the entire 12 s. If participants pressed the space bar, the picture was randomly assigned to either (1) leave the screen and be replaced with black screen until a total of 12 s had elapsed (success condition; approximately 50% of the press trials) or (2) remain on the screen until a total of 12 s had elapsed (failure condition; approximately 50% of the press trials). After 12 s had elapsed, participants were cued to select one of three reasons for pressing (*upset*, *bored*, *other*) or not pressing (*interested*, *indifferent*, *other*) the space bar (see **Figure 1**). The choice of these answer options was motivated by literature reviewed above, suggesting that people regulate their emotions when feeling distressed (e.g., Gross et al., 2006) and that viewing time might be related to boredom/interest in the picture (Lang et al., 1993). The option *other* was included so that participants did not have a sense of forced choice, and thus could note that they chose to end or not end picture presentation for reasons we did not anticipate based on previous research. This screen remained present until a response was recorded, at which point, participants were asked to provide an arousal rating on a scale ranging from 1 (*mildly arousing*) to 9 (*highly arousing*), indicating how the picture made them feel. The rating screen remained present until a response was recorded. Lastly, participants saw a black screen with a central white ellipsis that lasted 2 s to provide a brief break between trials.

#### Pretesting

We conducted pretesting of the picture task and stimuli with a separate sample of 15 Tufts undergraduates. The goals of pretesting were to (1) determine the ideal picture presentation duration and (2) identify areas of interest (AOIs) in each picture for subsequent analyses of the eye tracking data in the present study.

On the basis of pretesting, we selected 12 s as the picture duration because this duration when compared to three others (6, 8, 10 s) came closest to yielding a button press percentage of roughly 67%, enough to yield an approximately even number of pictures in the no-press, press-success, and press-failure cells of the design. In addition, when presented for 12 s, participants indicated that they pressed the space bar to terminate presentation of negative pictures most frequently because they were upset and least frequently because they were bored in comparison to other trial lengths. This suggested that button pressing in the 12-s context was more likely than in the context of other trial lengths to be motivated by a desire to regulate negative emotions, our key construct of interest.

Also in pretesting (using the subset of 13 participants for whom eye tracking data were available), we identified AOIs based on eye gaze fixations observed within the first 750 ms of picture presentation. Since all space bar presses occurred after 750 ms of presentation, the AOIs we identified captured spontaneous deployment of attention as opposed to attentional processes that were driven by the attempt to end the situation. The AOIs were polygon-shaped, and their sizes varied from one picture to the next. Up to three AOIs were retained for each picture, all of which had to meet the criterion of being based on at least 60% of the participants. There was no significant difference in the size of AOIs for negative (*M* = 5.13, *SD* = 2.67) compared to neutral (*M* = 4.60, *SD* = 2.60) pictures, *t*(58) = 0.78, *p* = 0.440, *dz* = 0.20 where AOI size was defined as the percentage of the picture area the AOI occupies. Visual inspection indicated that the AOIs generally reflected primary features of the scenes mostly in the center of the screen and captured all of the information in each picture that we judged to be either negative or neutral (as appropriate in light of normative ratings for these two categories of stimuli).

### DEPENDENT VARIABLES

In addition to subjective ratings of arousal, peripheral physiological data (corrugator activity, electrocardiography, and electrodermal activity) were collected continuously during the task using an MP150 system (Biopac, Goleta, CA, USA). These data were processed offline using ANSLAB (Wilhelm and Peyk, 2005). Bilateral eye-tracking data were also unobtrusively collected using a Tobii T120 Eye Tracker (Danderyd, Sweden; sampled at 60 Hz). These data were processed offline using Tobii Studio software. The details of these recordings and how we computed our dependent variables of interest are described below.

#### Button pressing behavior

The percentage of trials on which participants pressed the space bar was calculated within the negative and neutral picture categories. This score represented the frequency with which participants elected to end the situation. In addition, we calculated mean response time on trials on which the button was pressed within the negative and neutral picture categories. This score represented the speed with which participants elected to end the situation.

#### Ratings of arousal

Ratings of arousal were used to index subjective emotional experience. These values were averaged across trials separately for no-press, press-success, and press-failure conditions within the negative and neutral picture categories for each participant.

#### Corrugator electromyography

Corrugator electromyography was selected as an index of facial expressive behavior, even that which is not overtly observable. It has been shown to be sensitive to stimulus valence, exhibiting greater activity in response to negative stimuli and lower activity in response to pleasant stimuli (Bradley and Lang, 2007). Two 4 mm Ag/AgCl electrodes were placed in bipolar configuration over the left eye per Fridlund and Cacioppo (1986). Corrugator electromyography was sampled at 1000 Hz and bandpass-filtered online (5 Hz to 3 kHz; 60 Hz notch filter on). Offline, data were resampled to 400 Hz, rectified and smoothed with a 16-Hz low-pass filter, decimated to 4 Hz, and smoothed with a 1-s prior moving average filter.

Baseline-corrected corrugator activity was calculated for each 250 ms time bin after picture onset by subtracting the 250 ms time bin immediately preceding picture onset for each trial for each participant. After baseline correction, the data for this 12-s window of interest were averaged across time bins and trials separately for no-press, pre-press-success, post-press-success, pre-press-failure, and post-press-failure conditions within the negative and neutral picture categories for each participant. Separating pre-press from post-press values allowed us to assess for change in corrugator activity as a function of the attempt to end the situation by pressing the button.

#### Electrocardiography

Electrocardiography was used to measure HR, which is dually innervated by the sympathetic and parasympathetic branches of the autonomic nervous system. In event-related paradigms involving passive viewing of negative pictures, HR exhibits an initial, parasympathetically mediated deceleration (Bradley and Lang, 2007). Two disposable Ag/AgCl electrodes pregelled with 7% chloride gel (1 cm circular contact area) were placed under the left and right collarbones on the chest after swabbing with an alcohol or an electrode prep pad. ECG was acquired continuously at 1,000 Hz. Offline, the ECG signal was downsampled to 400 Hz and bandpass-filtered from 0.5 to 40 Hz.

Interbeat interval series were created by identifying R-spikes using automated ANSLAB algorithms. R-spikes that were not detected automatically, thus leading to an erroneously long period between successive R-spikes, were marked for inclusion by hand. Similarly, R-spikes that were identified incorrectly, thus leading to an erroneously short period between successive R-spikes, were removed by hand. Following such artifact correction, the interbeat interval series was converted to HR in beats per minute. HR data were decimated to 10 Hz and then smoothed with a 1-s prior moving average filter.

Baseline-corrected HR was calculated for each 100-ms time bin following picture onset by subtracting the 100-ms time bin immediately preceding picture onset for each trial for each participant. After baseline correction, the data for this 12-s window of interest were averaged across time bins and trials separately for no-press, pre-press-success, post-press-success, pre-press-failure, and post-press-failure conditions within the negative and neutral picture categories for each participant. Separating pre-press from post-press values allowed us to assess for change in HR as a function of the attempt to end the situation by pressing the button.

#### Electrodermal activity

Electrodermal activity (EDA) was selected as a pure measure of sympathetic activation of the autonomic nervous system. Two disposable Ag/AgCl electrodes pregelled with 0.5% chloride isotonic gel (1 cm circular contact area) were attached to the distal phalanges of the index and middle fingers on the left hand. EDA level was recorded with DC coupling and constant voltage electrode excitation at 31.25 Hz (sensitivity = 0.7 nS). Offline, EDA was smoothed with a 1 Hz, low-pass filter, decimated to 10 Hz, and linearly detrended on a trial-by-trial basis. One ground electrode for all physiological channels was placed on the forehead.

Baseline-corrected EDA was calculated for each 100-ms time bin following picture onset by subtracting the 100-ms time bin immediately preceding picture onset for each trial for each participant. After baseline correction, the data for this 12-s window of interest were averaged across time bins and trials separately for no-press, pre-press-success, post-press-success, pre-press-failure, and post-press-failure conditions within the negative and neutral picture categories for each participant. Separating pre-press from post-press values allowed us to assess for change in EDA as a function of the attempt to end the situation by pressing the button.

#### Eye tracking

Eye tracking was used to measure overt deployment of attention to key information in the pictures. This was operationalized as the amount of time participants spent looking within the pretest-defined AOIs for each picture relative to total viewing time on a second-by-second basis.

Fixations within the AOI were identified using the “Tobii fixation filter” algorithm, proposed by Olsson (2007). The algorithm uses eye tracker sample rate (60 Hz in our case) and the distance between neighboring gaze data points to calculate changes in the eye movement velocity for all eye movement samples. If the velocity is below 0.42 pixels/ms, the raw data points are assigned to the same fixation, but if it is above this threshold, they are assigned to a new fixation. To determine the location of the fixation, the algorithm uses the mean of the coordinates from the raw data points belonging to the same fixation (Tobii Technology, 2010, p. 78).

Fixation data for the 12-s window of interest were averaged across time and trials separately for no-press, pre-press-success, post-press-success, pre-press-failure, and post-press-failure conditions within the negative and neutral picture categories for each participant. Separating pre-press from post-press values allowed us to assess for change in looking time as a function of the attempt to end the situation by pressing the button.

### POST-TASK QUESTIONNAIRES

At the end of the study session, participants completed self-report questionnaires assessing exposure to stressors (Life Experiences Survey; Sarason et al., 1978), and the perceived probability and persistence of a list of emotional states (Emotion Reactivity and Perseveration Scale, modeled after the Anxiety Reactivity and Perseveration Scale; Rudaizky et al., 2012). They also provided demographic information and information about their horror movie watching habits (liking and frequency). The constructs measured in these questionnaires were included as possible moderators of observed effects; analyses using these measures are not reported in this paper.

### DATA RETENTION

To prevent leveraging of condition estimates by extreme, outlying values, only trials falling less than 4 SD from the within-subjects mean were retained on a measure-by-measure basis for each participant. In addition, to prevent leveraging of sample-wide differences between conditions, multivariate outliers were excluded [i.e., participants for whom the estimate of Mahalanobis Distance across variables within each measure was too large (*p* < 0.001); see Fidell and Tabachnick, 2003]. In addition, some participants failed to contribute data for all dependent measures due to artifacts and/or equipment issues. Due to listwise deletion of missing data, only participants who provided data in all conditions relevant to particular analyses were included in those analyses. As a result, degrees of freedom vary across statistical tests.

## RESULTS

### PRELIMINARY ANALYSES

#### Did the pictures produce the expected emotional response?

Our assumption in presenting negative and neutral pictures was that the negative pictures, more so than neutral pictures, would elicit negative emotions that people might be motivated to regulate by trying to end the situation. This idea would be confirmed by finding higher self-reported arousal, corrugator activity, EDA, or greater deceleration in HR during the 12-s time period after picture onset on negative trials relative to the neutral ones.

As expected, a paired samples *t*-test across all trials (regardless of whether or not they pressed the button) showed that participants experienced the negative pictures (*M = *4.62, *SD *= 1.31) as more intense than the neutral pictures (*M *= 2.12, *SD *= 0.77), *t*(57) = 16.78, *p *< 0.001, *dz* = 2.34, as reflected in self-reported arousal. Negative pictures also led to greater corrugator activity (*M = *1.01, *SD *= 1.94) than neutral pictures (*M *= 0.04, *SD *= 2), *t*(57) = 2.43, *p *= 0.018, *dz* = 0.49. There was a greater deceleration in HR for negative pictures (*M *= –3.56, *SD *= 2.14), compared with neutral pictures (*M *= –2.66, *SD *= 1.96), *t*(54) = –3.83, *p *< .001, *dz* = –0.44. Finally, EDA activity was greater for negative pictures (*M* = 0.07, *SD* = 0.14) than neutral pictures (*M* = 0.04, *SD* = 0.16), *t*(57) = 1.93, *p* = 0.058, *dz* = 0.19, a difference that was on the border of significance. Overall, these results strongly suggest that the pictures produced the expected emotional response.

#### Validating the areas of interest

Although proportion of time spent looking at the whole screen was equivalent in the negative (*M* = 0.79, *SD* = 0.17) and neutral conditions (*M* = 0.80, *SD *= 0.16), *t*(57) = -1.03, *p* = 0.307, *dz* = -0.06, people spent more time looking in negative AOIs (*M* = 0.20, *SD* = 0.06) than neutral AOIs (*M* = 0.19, *SD* = 0.05), *t*(57) = 3.06, *p* = 0.003, *dz* = 0.18. Thus, eye tracking data confirmed that the pretest-defined AOIs captured the intended distinction between negative and neutral as appropriate in light of normative ratings for these two categories of stimuli.

### HYPOTHESIS TESTING

#### Hypothesis 1. Do people end negative situations more frequently and faster than neutral situations?

To test our hypotheses that people would attempt to end the situation (as evidenced by choosing to press the space bar) more often and faster on negative than neutral trials, paired samples *t*-tests were used to compare percentage of space bar presses and RT for negative versus neutral trials. Contrary to what we expected, people did not press the space bar more frequently on negative (*M* = 44.83%, *SD* = 27.55) relative to neutral (*M* = 47.87%, *SD* = 29.88) trials, *t*(57) = -0.78, *p* = 0.441, *dz = *-0.12. Similarly, there was no significant difference in RT to press the space bar on the negative (*M* = 5.58 s, *SD* = 2.12) relative to neutral trials (*M* = 5.83 s, *SD* = 1.72), *t*(50) = -0.87, *p* = 0.390, *dz = *-0.13.

#### Hypothesis 2. Do people end negative situations because they are upset?

To test our hypotheses that people would report that they tried to end the situation because they were upset, paired samples *t*-tests were used to compare reasons for pressing the button on negative versus neutral trials. As hypothesized, people chose to press the space bar more frequently because they were upset by negative (*M* = 69.14%, *SD* = 26.86) relative to neutral pictures (*M* = 2.07%, *SD* = 5.38), *t*(50) = 18.29, *p* < 0.001, *dz* = 3.46.

We also analyzed button pressing frequency in terms of other reasons people gave for either pressing or not pressing the button. Perhaps not surprisingly, they chose to press the space bar more frequently because they were bored by neutral (*M* = 85.67%, *SD *= 18.19) relative to negative pictures (*M* = 17.26%, *SD* = 20.54), *t*(50) = –23.23, *p* < 0.001, *dz* = -3.53. In addition, people chose not to press the space bar more frequently because they were interested in negative (*M* = 76.26%, *SD* = 26.18) relative to neutral pictures (*M* = 62.2%, *SD* = 28.13), *t*(55) = 4.29, *p* < 0.001, *dz* = 0.52. By contrast, they chose not to press the space bar more frequently because they were indifferent to the neutral (*M* = 33.19%, *SD* = 26.33) relative to the negative pictures (*M* = 10.7%, *SD* = 16.09), *t*(55) = -6.23, *p* < 0.001, *dz* = -1.03.

#### Revisiting Hypothesis 1. Could the frequency of ending negative relative to neutral situations be moderated by arousal?

Summarizing the results of testing Hypotheses 1 and 2 above, we found that people attempted to end the situation more frequently when they were upset (negative situation) or bored (neutral situation), and they elected not to end the situation more frequently when they were interested (negative situation) or indifferent (neutral situation). However, there was neither a difference in the percentage of button presses nor in the speed with which the button was pressed between the negative and neutral conditions. Since the reasons participants provided suggested that they were, in fact, attempting to regulate distress and boredom, we suspected that comparing negative to neutral picture categories – which encapsulate variation in both valence and arousal – may be too broad; it may be that the behavioral differences of interest are sensitive to just one of these dimensions.

As such, we examined whether the frequency and speed of attempts to end the situation varied as a function of arousal within the negative and neutral categories. To test this, we established high-arousal and low-arousal subsets based on a median split of the normative IAPS arousal ratings within the negative and neutral picture categories. In our stimulus set, the median arousal was 5.79 for the negative pictures and 3.42 for the neutral pictures. This median split procedure yielded 4 cells each comprised of 15 pictures as follows: high-arousal negative, low-arousal negative, high-arousal neutral, and low-arousal neutral. Note that because the arousal cut-off is different for the two negative and neutral picture categories, we do not treat arousal as a two-level factor in subsequent analyses. Instead, we use paired *t*-tests to compare high-arousal to low-arousal conditions within the negative and neutral categories.

Within the negative condition, the percentage of space bar presses was higher for high-arousal (*M* = 57.69%, *SD* = 28.62) than low-arousal pictures (*M* = 40.77%, *SD* = 28.18), *t*(51) = 4.7, *p* < 0.001, *dz* = 0.6. Within the neutral condition, by contrast, the percentage of presses was *lower* for high-arousal (*M* = 49.38%, *SD* = 26.35) than low-arousal pictures (*M* = 60.41%, *SD* = 26.05), *t*(48) = -6.36, *p* < 0.001, *dz* = -0.42. A similar pattern was evident for response time. Within the negative condition, RT (in seconds) was faster for high-arousal (*M* = 5.04, *SD *= 2.71) than low-arousal (*M* = 5.83, *SD* = 2.17) pictures, *t*(51) = -3.28, *p* = 0.002, *dz* = -0.32. Within the neutral condition, RT was *slower* for high-arousal (*M* = 6.14, *SD* = 1.9) than low-arousal (*M *= 5.41, *SD* = 1.77) pictures, *t*(48) = 3.29, *p* = 0.002, *dz* = 0.4.

**FIGURE 2:**
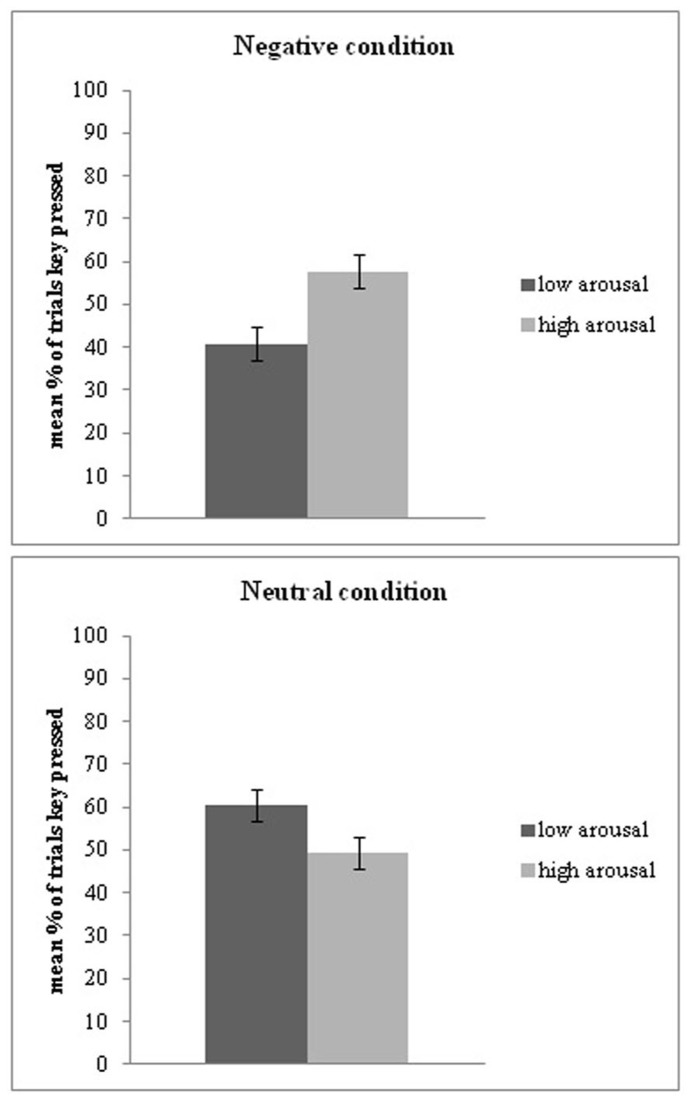
**Mean percentage of trials when the space bar was pressed is reported separately for negative and neutral pictures, with either low or high-arousal.** People pressed the space bar significantly more for high-arousal negative pictures relative to low-arousal negative pictures. Within the neutral pictures, people pressed the space bar significantly more often for low-arousal in comparison to high-arousal pictures. Error bars reflect ±1 SEM.

These results indicate that participants attempted to end high-arousal negative and low-arousal neutral situations more frequently and more rapidly than they attempted to end low-arousal negative and high-arousal neutral situations, respectively (see **Figure 2**).

#### Revisiting Hypothesis 2. Could people’s choice to end negative situations because they are upset be moderated by arousal too?

Consistent with the above pattern for button-pressing behavior, reasons for pressing the space bar were moderated by arousal too. Specifically, within the negative condition, people chose to press the space bar because they were upset more for high-arousal (*M* = 84.84%, *SD* = 25.75) than low-arousal (*M *= 53.32%, *SD* = 35.59) pictures, *t*(51) = 6.91, *p* < 0.001, *dz* = 1.01. By contrast, within the neutral condition, people chose to press the space bar because they were upset to a similar degree for high-arousal (*M* = 3.02%, *SD* = 8.89) and low-arousal (*M* = 1.29%, *SD* = 4.94) pictures, *t*(48) = 1.302, *p* = 0.199.

**FIGURE 3:**
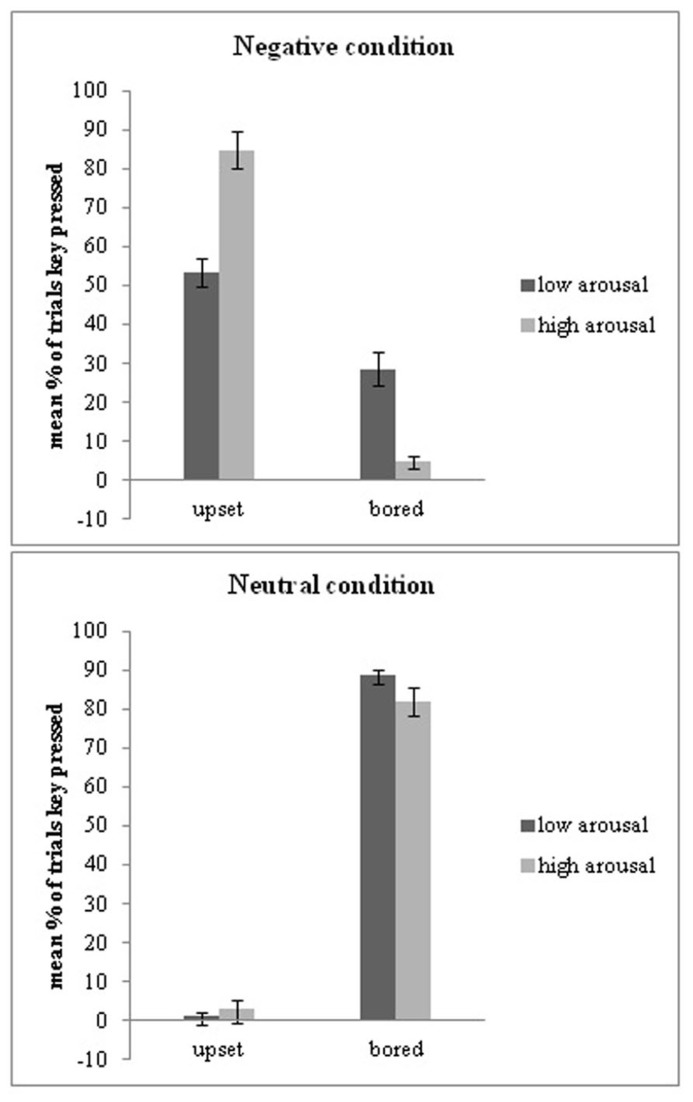
**Differences in reasons people provide for pressing the space bar are reported separately for negative and neutral pictures, with either low or high-arousal.** People report being more upset by the high-arousal negative pictures, and more bored by the low-arousal both negative and neutral pictures. Error bars reflect ±1 SEM.

Furthermore, within the negative condition, they chose to press the space bar *less* because they were bored by high-arousal (*M* = 4.68%, *SD* = 1.22) than low-arousal (*M* = 28.64%, *SD *= 32.03) pictures, *t*(51) = -6.06, *p* < 0.001, *dz* = -1.06. Within the neutral condition, they pressed the button less because they were bored by high-arousal (*M* = 81.89%, *SD* = 24.5) than low-arousal (*M* = 88.67%, *SD* = 15.94) pictures, *t*(48) = -2.418, *p* = 0.019, *dz* = 0.33 (see **Figure 3**).

Finally, considering the choice to remain in the given situation, within the negative condition, people chose not to press the space bar more because they were interested in high-arousal (*M* = 81.27%, *SD* = 30.56) than low-arousal (*M* = 72.67%, *SD* = 29.42) pictures, *t*(50) = 2.18, *p* = 0.034, *dz* = 0.29. In contrast, they chose not to press the space bar *less* because they were indifferent to high-arousal (*M* = 6.08%, *SD* = 20.18) than low-arousal (*M* = 15.5%, *SD* = 18.4) pictures, *t*(50) = -3.32, *p* = 0.002, *dz* = -0.49. This pattern was echoed within the neutral condition, in which people chose not to press the space bar more because they were interested in high-arousal (*M* = 70.49%, *SD* = 26.57) than low-arousal (*M* = 55.93%, *SD* = 31.45) pictures, *t*(52) = 4.65, *p* < 0.001, *dz* = 0.5. They also chose not to press the space bar *less* because they were indifferent to the high-arousal (*M* = 25.66%, *SD* = 23.93) than low-arousal (*M* = 38.2%, *SD* = 29.47) pictures, *t*(52) = -4.21, *p* < 0.001, *dz* = 0.47.

Overall, these results suggest that participants attempted to end high-arousal negative situations more than low-arousal negative situations when they were upset. Regardless of whether the situation was negative or neutral, participants attempted to end low-arousal situations more than high-arousal situations when they were bored, they remained in high-arousal situations more than low-arousal situations when they were interested, and they remained in high-arousal situations less than low-arousal situations when they were indifferent.

#### Hypothesis 3a. Does ER failure increase negative emotions relative to ER success?

Since our analyses so far showed that attempts to end the situation were made most frequently for high-arousal negative and low-arousal neutral pictures, we focused our test of Hypothesis 3a on trials in which participants pressed the button to end the situation in these two cells. Specifically, we tested whether there was a pre-to-post-press increase in emotional responding for the success versus failure conditions. For this purpose, we conducted repeated measures GLMs with two factors, time (pre-press, post-press) and manipulated outcome (success, failure) for the physiological measures and paired samples *t*-tests for the ratings of arousal (for which we only had post-press data).

Contrary to Hypothesis 3a, GLM analyses revealed no significant interactive effects of time and manipulated outcome on corrugator activity, HR, EDA, or ratings of arousal for the high-arousal negative condition. A similar absence of effects was observed for the low-arousal neutral condition. In that case, however, there was one significant difference. Namely, ratings of arousal were lower for the success condition than for the failure condition on low-arousal neutral trials. These results are summarized in **Table 1**. Overall, they suggest that ER failure did not increase negative emotions relative to ER success.

**Table 1:** Mean (and Standard Deviations) for the dependent measures as a function of manipulated outcome and time in high-arousal negative and low-arousal neutral conditions.

Statistics		Failure		Success	
		Pre	Post	Pre	Post
**High-arousal negative**
Corrugator activity	*F*(1,43) = 1.5, *p* = 0.227	–0.14 (12.49)	0.32 (11.81)	1.92 (3.97)	1.38 (6.03)
Heart rate	*F*(1,41) = 3.08, *p* = 0.087	–2.68 (3.20)	–3.28 (3.49)	–3.03 (3.55)	–3.04 (3.90)
Electrodermal activity	*F*(1,43) = 0.40, *p* = 0.531	0.05 (0.26)	0.14 (0.29)	0.00 (0.20)	0.14 (0.29)
Ratings of arousal	*t*(46) = 1.12, *p* = 0.267	–	5.75 (1.57)	–	5.91 (1.62)
Looking time in the AOIs	*F*(1,46) = 0.16, *p* = 0.690	0.26 (0.11)	0.01 (0.03)	0.27 (0.13)	0.01 (0.03)
**Low-arousal neutral**
Corrugator activity	*F*(1,44) = 1.31, *p* = 0.259	–0.66 (2.97)	–0.32 (1.78)	0.06 (1.14)	–0.23 (1.48)
Heart rate	*F*(1,41) = 1.58, *p* = 0.215	–3.28 (3.43)	–3.18 (2.89)	–3.09 (3.55)	–2.29 (3.75)
Electrodermal activity	*F*(1,44) = 1.4, *p* = 0.244	0.04 (0.19)	0.04 (0.13)	0.07 (0.24)	0.05 (0.15)
Ratings of arousal	*t*(44) = -2.29, *p* = 0.027	–	1.9 (0.93)	–	1.62 (0.66)
Looking time in the AOIs	*F*(1,44) = 0.57, *p* = 0.454	0.27 (0.10)	0.01 (0.01)	0.26 (0.12)	0.01 (0.01)

*The *F* statistics reported above are for the time (pre-press, post-press) × manipulated outcome (failure, success) interaction.*

#### Hypothesis 3b. Do people use attentional deployment to compensate for ER failure?

The absence of outcome differences in emotional responding might suggest that participants were compensating for the failure of the space bar press to end the situation by using one or more alternative ER strategies. In this study, we addressed this possibility by focusing specifically on attentional deployment in the high-arousal negative and low-arousal neutral conditions. Contrary to Hypothesis 3b, GLM analyses assessing effects of time (pre-press, post-press) and manipulated outcome (success, failure) showed that looking time in the AOIs decreased from pre-to-post space bar press for both high-arousal negative *F*(1,46) = 307.41, *p* < 0.001 and low-arousal neutral picture *F*(1,46) = 357.70, *p* < 0.001, and it did so to the same extent in the success and failure conditions. These results are summarized in **Table 1**. Overall, they suggest that participants did not use attentional deployment to compensate for ER failure.

#### Secondary analyses. Is there a reduction in frequency of button press across time?

The absence of an impact of manipulated regulatory outcome (success versus failure) on both emotional responding and compensatory attentional deployment led us to consider the possibility that participants were insufficiently invested in the task and, in particular, progressively less motivated to press the space bar as they learned (implicitly or explicitly) that the button press had only a 50:50 chance of having the intended effect. We thus examined whether the percentage of button presses decreased from the first to the second block of the picture task. Indeed, a paired samples *t*-test revealed a modest but significant drop in the frequency of button presses in block 2 (*M* = 44.15%, *SD* = 24.73) relative to block 1 (*M* = 48.32%, *SD* = 26.25), *t*(56) = 2.55, *p* = 0.014, *dz* = 0.16. However, this drop was found only for low-arousal negative pictures ( block 1 *M* = 41.19%, *SD* = 32.03; block 2 *M* = 33.98, *SD* = 32.03), *t*(56) = 2.36, *p* = 0.022, *dz* = 0.23, and for high-arousal neutral pictures (block 1 *M* = 45.69%, *SD *= 32.64; block 2 *M *= 38.64, *SD* = 32.17), *t*(56) = 2.14, *p* = 0.037, *dz* = 0.22. There was no signifcant drop in button pressing for high-arousal negative and low-arousal neutral pictures, both *p’*s > 0.05, the two conditions of central importance to testing Hypothesis 3.

## DISCUSSION

Our results show that participants attempted to end the situation more often for high-arousal than low-arousal negative pictures and more often for low-arousal than high-arousal neutral pictures. They reported doing so because they were upset or bored, respectively. Partially supporting our first two hypotheses, these observations suggest that people engage in situation-targeted ER strategies in order to alter their emotional experience when given an explicit opportunity to do so. Contrary to our first hypothesis, however, participants did not generally try to end negative situations more often or faster than neutral situations. Rather, differences in the use of situation-targeted ER strategies between negative and neutral situations were only evident in ways that depended on arousal level, as described above. Contrary to our third hypothesis, we found little to no evidence that failed attempts to end the situation increased emotional responding or prompted compensatory ER using attentional deployment.

In light of the lack of evidence for increased emotional responding or compensatory ER, we wondered if participant motivation flagged as the task wore on. Indeed, consistent with this possibility, we confirmed that button pressing waned in the second half of the task. However, this reduction in frequency of button presses was significant only for low-arousal negative and high-arousal neutral trials, the conditions for which people were not pressing the space bar frequently to begin with. Presumably this is because low-arousal negative and high-arousal neutral trials were insufficiently boring to motivate an attempt to end the situation or sufficiently interesting to motivate staying in the situation, respectively. This might suggest that when people are strongly motivated (as in the high-arousal negative and low-arousal neutral conditions), the frequency with which they attempt to regulate their emotions is immune to lack of control over the likely outcome of regulation.

### LINKS TO EXISTING LITERATURE

Gross et al. (2006) reported finding little evidence of two situation-targeted ER strategies, situation selection and situation modification, in everyday life. In that work, the authors used semi-structured interviews in which respondents were prompted to think of a time in the past couple of weeks when they tried altering their emotions. Since it might not always be clear to people that they are avoiding certain situations in order to regulate their emotions, the incidence of situation-targeted ER strategies may actually be higher than reported. In the present study, button pressing behavior indexed the frequency and speed of ER in which the goal was to end the situation to alter negative emotion. We found that in high-arousal negative and low-arousal neutral situations, people used a situation-targeted ER strategy roughly 60% of the time. We are hard-pressed to definitively categorize the button press as situation selection or situation modification. If one thinks of the button press as selecting a new, unemotional situation, situation selection may be the correct label. On the other hand, if one thinks of the button press as choosing to change the existing emotional situation, situation modification may be the correct label. Either way, these results give the impression that situation-targeted strategies may occur with greater frequency than was evident in interviews.

Previous research has shown that people choose different ER strategies based on the intensity of negatively valenced situations. Namely, in low-intensity negative situations, people choose reappraisal over distraction, while in high-intensity negative situations, people choose distraction over reappraisal (Sheppes et al., 2011). Echoing Sheppes et al. (2011), our results indicate that attempts to end emotion-eliciting situations also depend on intensity. We found that people pressed the space bar more frequently for high-arousal negative and low-arousal neutral pictures, and they did so because they were upset by the negative pictures and bored by the neutral pictures. Furthermore, people pressed the space bar faster on high-arousal negative trials, and low-arousal neutral trials. These results thus extend the idea that choice of ER strategy depends on situation intensity from a context in which participants chose between attentional deployment (distraction) and cognitive change (reappraisal; Sheppes et al., 2011) to a context in which a situation-targeted ER strategy was readily available. Revisiting an earlier point, had Gross et al.’s (2006) ER interview asked participants to consider their use of ER strategies to alter intense emotions specifically, situation-targeted ER strategies (situation selection, situation modification) may have been endorsed more frequently.

Our results also help shed light on the links between valence, arousal, and viewing time, as demostrated in Lang et al. (1993). These authors showed that viewing time was longest for pictures that were ranked most pleasant and most unpleasant relative to neutral. This quadratic relationship disappeared, however, when controlling for arousal. This suggests that the viewing time effect was explained by arousal. Indeed, in that study, people looked at high-arousal pictures longer than low-arousal pictures, an effect that remained even when they controlled for valence. Our results underscore the importance of arousal in explaining viewing time but add an important caveat. Namely, the effect of arousal on viewing time (as indexed by RTs to press the button) can be moderated by valence. In the present study, people spent less time viewing high-arousal negative and low-arousal neutral contents relative to low-arousal negative and high-arousal neutral contents, respectively. According to our results, it seems that there is a “tipping point” on the arousal scale at which a certain level of arousal gets to be “too much” or “too little,” which prompts people to decide to try to end the situation.

### THEORETICAL AND CLINICAL IMPLICATIONS

The SOC-ER framework suggests that we select and optimize ER strategies and, when our emotion-regulatory maneuvers do not successfully alter our emotions as intended, we can compensate by employing alternative strategies (Urry and Gross, 2010; Opitz et al., 2012). We found little evidence that failed attempts to end emotion-eliciting situations increased emotional responding relative to successful attempts. This might suggest that participants compensated for this ER failure by selecting an alternative ER strategy, which would be consistent with SOC-ER. However, we found no evidence to suggest that they did so using attentional deployment. If compensation occurred, it may have been by way of alternative ER strategies that we did not assess.

It is also possible that compensation in the wake of ER failure (having high-arousal negative and low-arousal neutral pictures remain on screen despite an avowed desire for them to go away) did not work the way we expected. It may be that, even when the key press successfully removed the picture from the screen, there was a tendency to maintain a mental representation of that content (e.g., via visual imagery and/or internal verbal behavior, both of which would constitute a form of rumination). If true, the success and failure conditions, though distinct in terms of objective input, were indistinct in terms of mental representation; in effect, compensation was occurring, as predicted by SOC-ER, but in the “wrong” direction. That is, instead of using alternate ER strategies to reduce negative emotion (attentional deployment) in the failure condition, subjects may have been using alternate ER strategies that increase negative emotion (rumination) in the success condition. We had no measure of rumination during the task thus this remains speculative.

Suri et al. (2013) argue that attentional deployment and CR are “most unambiguously cognitive in nature” (p. 198), unlike situation selection and situation modification, which are behavioral in nature. We wholeheartedly agree that attentional deployment and CR are “more cognitive” than situation-targeted strategies. Nevertheless, we would like to add that the latter still are cognitive to some degree. Specifically, ending an upsetting or boring situation by leaving (or pressing the button in this study) emerges as the result of a decision making process. Thus, to the extent that decision making constitutes a cognitive process, cognition is actually present in all five of the ER families described in Gross’s (1998) process model of ER.

In this study, we obtained evidence that people attempted to regulate boredom when viewing low-arousal neutral pictures. In addition, it was only for low-arousal neutral pictures that we saw any evidence that ER failure resulted in a stronger emotional response compared to ER success. Specifically, people felt more aroused when the pictures stayed on the screen compared to when they went away. It is worth noting here that investigators, including us, often say they do not pair the ER instructions with neutral pictures because there is no emotion to regulate (e.g., Urry, 2010). The present results suggest this is not strictly true, at least not for the least arousing neutral stimuli. For those stimuli, boredom is prominent and, according to our button press results, this is an emotional state that people are motivated to regulate. Future studies that examine regulation of boredom are warranted. Boredom is, afterall, an emotional state that may lead to troublesome behavior (Sommers and Vodanovich, 2000; Dahlen et al., 2004; Wegner et al., 2008). As such, learning whether people can regulate boredom could have clinical utility. In these efforts, it would be worth determining whether particular forms of ER are better suited than others for regulating boredom in low-arousal situations.

Avoidance behavior, particularly experiential avoidance, defined as a person’s unwillingness to experience negative emotions, sensations, feelings, and thoughts, and desire to change the form or frequency of situations giving rise to those experiences, is an example of ER through situation selection (Hayes et al., 1996). Hayes et al. (1996) argue that unhealthy avoidance of emotions can be implicated in many behavioral disorders such as substance abuse and dependence, obsessive-compulsive disorder (OCD), panic disorder with agoraphobia, and borderline personality disorder, also proposing that suicide might be the ultimate avoidance strategy. Thus, studying which situations people choose to approach, avoid, or modify, how frequently,in which contexts, and the cognitive, emotional, and social consequences of doing so can inform clinical practice and help in the development of appropriate therapy. The present study is a step toward meeting those goals.

It is important to note here that all families of ER, situation-targeted forms included, can be helpful and/or harmful depending on one’s goals and the context at hand. Always selecting situations to avoid negative emotions is unlikely to be helpful in the long run. Flexible deployment of ER processes appropriate for the present circumstances – e.g., sometimes approaching, sometimes avoiding emotion-eliciting situations – is apt to be key to adaptive functioning (e.g., Bonanno, 2013).

### LIMITATIONS OF THE CURRENT RESEARCH

Our approach to the questions of interest in this work has the benefit of contributing a novel and simple laboratory task to assess the use of situation-targeted ER strategies in a way that bypasses self-report methods. This contribution is significant because, although situation-targeted strategies are of central importance to the process model (Gross, 1998), studies that examine situation-targeted strategies are sorely lacking in the current literature (Webb et al., 2012). That being said, our approach also has several key limitations, three of which we discuss below.

One important limitation of this design is the lack of a success/failure manipulation in the no-press condition. This would have allowed us to determine to what extent agency, i.e., the perception that one is in control, plays a role in responses to the button press. In addition, to enhance the sense of agency, it would be useful to change the distribution of failed to successful attempts to end the situation. In the present work, there was a roughly even distribution such that the button press had the intended effect 50% of the time. An uneven distribution, in which the button press has the intended effect most (but not all) of the time would perhaps better mirror emotion regulatory efforts in everyday life, in which we often succeed in selecting or altering the situation as intended. Methodologically, this might also make participants more invested in the task.

Our study demonstated the importance of arousal in the decision to end emotion-eliciting situations. These results tie in with Lang et al. (1993), who showed that arousal ratings were correlated with viewing time. The present study was limited in this regard, however, because it was not possible to use a universal cut-off to define high- and low-arousal categories in our set of negative and neutral pictures. Had we used a universal cut-off to define high and low arousal, we would have had too few stimuli in the low-arousal negative and high-arousal neutral categories to achieve reliable estimates. This is because valence and arousal were not orthogonal; the negative category was defined *a priori* as unpleasant and high-arousal and the neutral category as neither pleasant nor unpleasant and low-arousal. Thus, in future studies, it would be useful to select stimuli using a single criterion of arousal to establish four comparable sets of stimuli in equal numbers: high-arousal negative, high-arousal neutral, low-arousal negative, and low-arousal neutral pictures. This approach would have enabled us to treat arousal and valence as orthogonal factors in GLM analyses.

Finally, this study was limited in its assessment of alternative ER strategies that participants may have used to compensate in the ER failure condition. Although attentional deployment was a well-considered candidate for compensatory ER, it was the only alternative we assessed. As noted previously, it is possible that compensation occurred via other means, e.g., rumination. Other alternatives are worth considering too. It might be useful, for example, to assess CR as another potential strategy of choice for compensation. CR is one of the most widely studied ER strategies, and people report using it frequently in everyday life. Gross et al. (2006), for example, found CR in 33% of emotional episodes reported by their participants. Additionally, there is evidence that attentional depoloyment is used together with other ER strategies, and differently at that – people tend to look away from emotional aspects of the picture more if they also used suppression, relative to reappraisal (Bebko et al., 2011). Using a variant of the picture task described here, we could ask participants at the end of each trial to indicate the strategies they used to alter their emotions and then compare the failure and success conditions. Importantly, in light of our earlier speculation about rumination, such efforts should measure ER strategies that would reduce emotional responding (of relevance in the failure condition) as well as ER strategies that might increase it (of relevance in the success condition).

### ADDITIONAL DIRECTIONS FOR FUTURE RESEARCH

This work suggests a number of promising broad directions for future research, three of which we consider here.

First, one important question to ask is how the effects we observed in this study vary in people who are younger or older than those studied herein. On the younger side of the coin, we know that adolescents’ emotional life is quite different than adults’: adolescents generally report feeling more emotion than adults in their everyday life, particularly negative emotion (Larson and Lampman-Petraitis, 1989; Larson et al., 2002; Weinstein et al., 2007; Riediger et al., 2009). This effect might partially be explained by use of different ER strategies and/or a different set of resources available for ER at different points in the life span (Opitz et al., 2012). On the older side of the coin, there is evidence suggesting that older adults experience more positive (Stawski et al., 2008) and less negative affect (Charles et al., 2001) than younger adults, which could be at least partially explained by their ER abilities (Urry and Gross, 2010). Furthermore, unlike older adults, younger adults sometimes actually feel better by deploying their attention to the negative aspects of the situation (Isaacowitz and Noh, 2011). Older adults are also more successful than younger adults in using attentional deployment and positive reappraisal (Lohani and Isaacowitz, 2013). In light of these findings, it seems likely that the frequency and success of situation-targeted ER strategies, and the contexts in which people use them vary across the life span. As such, there may also be differences in the ways in which people of different ages compensate for ER failure.

Second, although we are confident that this study is well suited to address our primary goals, our study sample is fairly homogenous (college undergraduates, mostly women, mostly Caucasian). As such, there may be interesting gender and/or cultural differences in situation-targeted ER that we will be unable to capture ideally in this work. For instance, evidence suggests that men use suppression more than women (Gross and John, 2003). In addition, research shows that European Americans use suppression less than Americans with an ethnic minority status (Gross and John, 2003). In other work, Matsumoto et al. (2008) found that cultures that value social order tend to suppress emotion more often and to show positively correlated reappraisal and suppression use. In contrast, cultures that value affective autonomy more than social order suppress emotion less often and show negatively correlated reappraisal and suppression use. There is also evidence suggesting that sociocultural contexts have a powerful influence on antecedent-focused automatic ER, which is defined as changes to one’s emotions without consciously deciding to do so, without attending to this regulatory process, and without engaging in deliberate control (Mauss et al., 2008). Future work should draw on broad community populations to obtain culturally diverse samples with even gender distributions.

Finally, in the laboratory task developed in present research, we explicitly instructed people to end the situation if they wanted. In addition, by asking about their reasons for ending the situation, we explicitly cued them to consider that they might do so if they became upset or bored. In everyday contexts, such explicit cues regarding opportunities and reasons for regulating one’s emotions are rare. In that case, what we observed in this task may be an overestimate of the use of situation-targeted strategies in daily life. Thus, another line of research should examine situation-targeted strategies in situations with greater external validity. One way to go about it would be to expose subjects to situations in the lab that are closer to real-world experiences, e.g., assessing how frequently they choose whether they want to have a potentially unpleasant/anxiety-provoking task or personal interaction, and under what circumstances they make this situation-targeted decision. Another alternative is to use methods in the real world, such as experience sampling to gauge situation-targeted ER in the context of very distressing and very boring life events. Such efforts have promise in yielding even better estimates of the use of situation-targeted ER strategies in daily life.

## CONCLUSION

In sum, we sought to determine how frequently and for what reasons people would use readily available situation-targeted strategies to regulate their emotions. We also sought to test the notion of compensatory ER as proposed in the SOC-ER framework. To accomplish these goals, we developed a novel laboratory task in which participants decided whether or not to end negative and neutral situations and indicated the reasons for their choices. We manipulated the outcome of these decisions such that some situations ended successfully as intended while other situations failed to end. Our results suggest that people use situation-targeted strategies to regulate their emotions, especially in high-arousal negative situations when they are upset and in low-arousal neutral situations when they are bored. We observed no evidence that people experience more negative emotions when their attempt at situation-targeted ER failed to have the intended effect. We also observed no evidence that people compensate using attentional deployment in this scenario. There are a number of important directions for future research in this domain. Should these findings replicate, they have important theoretical and clinical implications. Overall, the current study provides an experimental test of aspects of the SOC-ER framework and contributes a novel and simple laboratory task to assess the use of situation-targeted ER strategies.

## AUTHOR CONTRIBUTIONS

Lara Vujovic and Heather L. Urry conceived and designed the study, with significant input from Philipp C. Opitz and Jeffrey L. Birk. Lara Vujovic collected most of the data. Lara Vujovic and Heather L. Urry analyzed the data with input from Philipp C. Opitz and Jeffrey L. Birk. Lara Vujovic, Heather L. Urry, Philipp C. Opitz, and Jeffrey L. Birk wrote the manuscript.

## Conflict of Interest Statement

The authors declare that the research was conducted in the absence of any commercial or financial relationships that could be construed as a potential conflict of interest.

## Footnotes

1 The following pictures from the International Affective Picture System (Lang et al., 2008) set, listed by catalog number, were used: negative – 2301, 2312, 2590, 2661, 2683, 2710, 2718, 2795, 3001, 3016, 3060, 3195, 3212, 3280, 3500, 3530, 6315, 6570.1, 6825, 8230, 8231, 8485, 9046, 9075, 9220, 9252, 9254, 9265, and 9419; neutral – 2026, 2036, 2101, 2102, 2104, 2107, 2190, 2191, 2200, 2235, 2359, 2377, 2382, 2383, 2393, 2396, 2397, 2411, 2512, 2521, 2575, 2593, 2595, 2597, 2745.1, 2870, 7493, 7496, 7550, and 7632.
